# P-849. Evaluation of Interactive Provider Dashboards for Comparison of Outpatient Antibiotic Prescribing for Respiratory and Otic Conditions in Urgent and Quick Care Clinics

**DOI:** 10.1093/ofid/ofaf695.1057

**Published:** 2026-01-11

**Authors:** Kelly M Percival, Kimberly C Dukes, Gosia Clore, Stacey Hockett-Sherlock, Dilek Ince, Mary Vaughan-Sarrazin, Nathan Shaw, Daniel J Livorsi

**Affiliations:** University of Iowa Health Care, Iowa City, IA; University of Iowa Carver College of Medicine, Iowa City, Iowa; University of Iowa, Iowa City, Iowa; University of Iowa, Iowa City, Iowa; University of Iowa Hospitals & Clinics, Iowa City, Iowa; University of Iowa College of Medicine, Iowa City, Iowa; University of Iowa Carver College of Medicine, Iowa City, Iowa; University of Iowa Carver College of Medicine, Iowa City, Iowa

## Abstract

**Background:**

Antibiotic overuse for viral respiratory conditions that never require antibiotics is common in the outpatient setting, especially walk-in clinics. We evaluated the effect of a multifaceted stewardship intervention, which included sending providers individualized peer comparison feedback reports on their antibiotic use for conditions that do not benefit from antibiotics (“never-events”).

**Figure:**
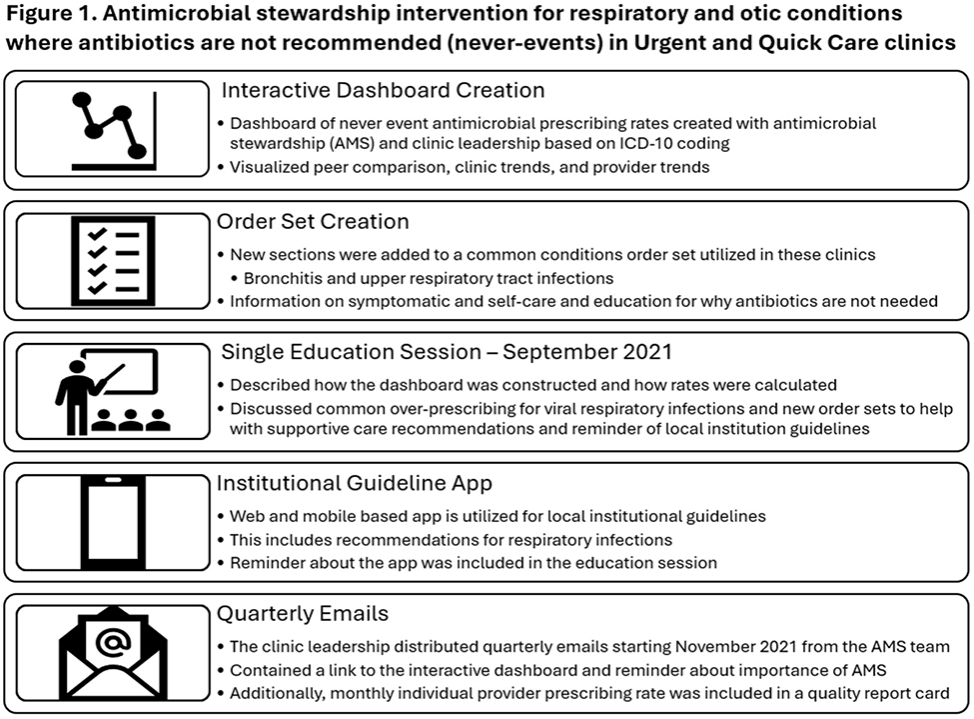

**Figure:**
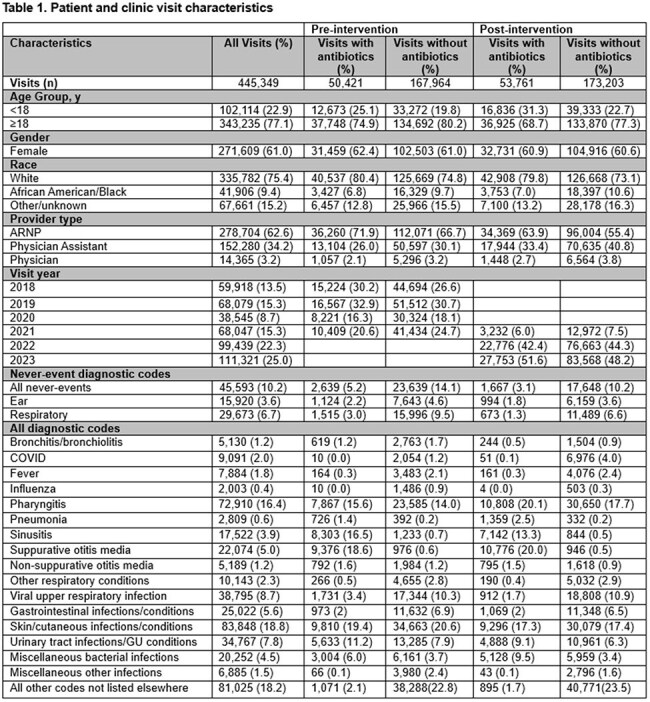

**Methods:**

We used mixed-methods to evaluate the intervention (Figure 1) in 8 walk-in clinics during a baseline period (Jan 2018-Oct 2021) and an intervention period (Nov 2021-Dec 2023). To analyze whether the intervention was associated with changes in antibiotic-prescribing across all visits (regardless of the diagnosis), we fit a generalized linear mixed model using a Poisson distribution and a log link, including random intercepts for physicians and adjustment for practice changes that occurred during the COVID-19 pandemic. Secondary outcomes included changes in the use of never-event diagnostic codes. In 2023, we conducted 17 semi-structured interviews with 10 providers about the acceptability of the metric in comparison to a new metric for all respiratory diagnoses.

**Figure:**
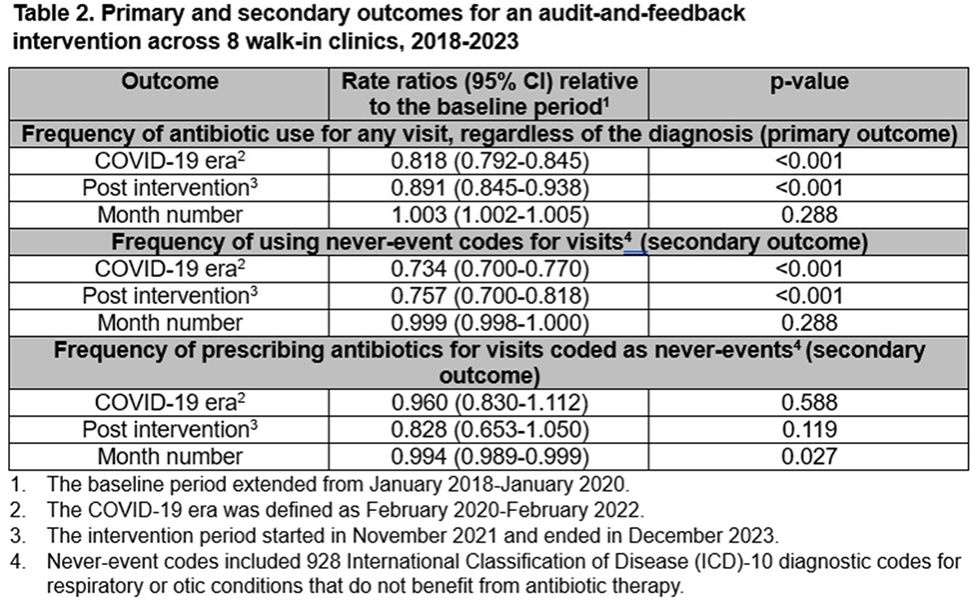

**Figure:**
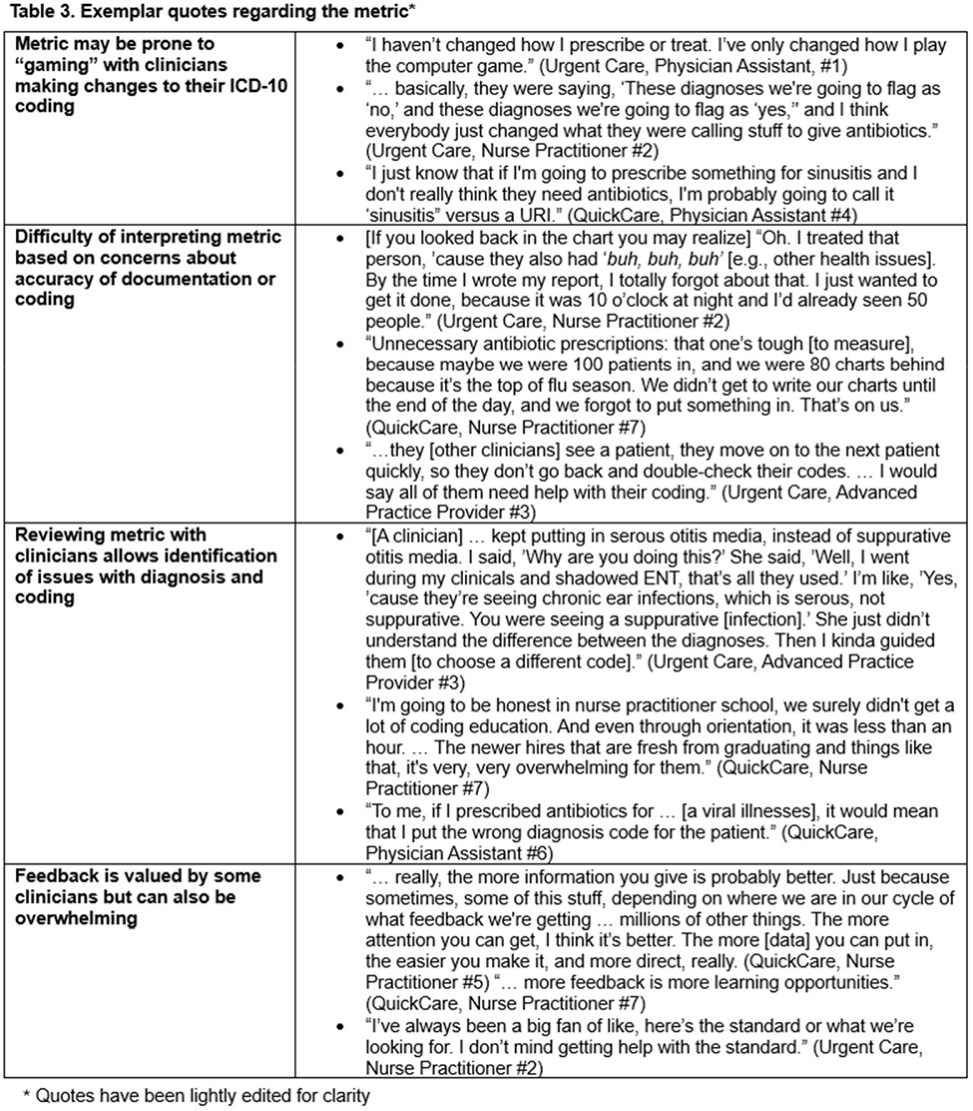

**Results:**

There were 445,349 visits; median age was 27 (IQR 18-44), and 61% were female (Table 1). After implementation of the intervention, the frequency of antibiotic-prescribing for all visits changed from 23.1% to 23.7% (p< 0.001), and the use of never-event codes changed from 12.0% to 8.5% (p< 0.001). In the adjusted analysis, the frequency of antibiotic-prescribing across all visits was 11% lower compared to before the intervention (RR 0.89, 95% CI 0.85-0.94), and the use of never-event diagnostic codes decreased by 24% relative to baseline (RR 0.76, 95% CI 0.70-0.82) (Table 2). Some providers valued receiving feedback on the metric while others admitted to shifting their codes to avoid metric detection (Table 3).

**Conclusion:**

Delivering feedback to walk-in clinics providers on their antibiotic use was associated with reductions in antibiotic-prescribing across all visits but also changes in diagnostic coding (i.e., “gaming”). Antibiotic stewardship programs should monitor for changes in both when implementing new outpatient metrics.

**Disclosures:**

All Authors: No reported disclosures

